# The Modulatory Effect of an Ethanolic Extract of *Anredera cordifolia* (Ten.) on the Proliferation and Migration of Hyperglycemic Fibroblasts in an *In Vitro* Diabetic Wound Model

**DOI:** 10.1155/2024/2812290

**Published:** 2024-10-08

**Authors:** Elisa Vanessa Heisler, Bárbara Osmarim Turra, Nathália Cardoso de Afonso Bonotto, Ivana Beatrice Mânica da Cruz, Marco Aurélio Echart Montano, Verônica Farina Azzolin, Jacir Dal Magro, Felipe Zaniol, Juliano Perottoni, Maria Eduarda Chelotti, Fernanda dos Santos Trombini, Ednea A. Maia-Ribeiro, Fernanda Barbisan, Maria Denise Schimith

**Affiliations:** ^1^Nursing Department, Federal University of Santa Maria, Santa Maria, Brazil; ^2^Federal University of Santa Maria, Santa Maria, Brazil; ^3^Technologist in Aesthetics and Cosmetology, Federal University of Santa Maria, Santa Maria, Brazil; ^4^Department of Morphology, Federal University of Santa Maria, Santa Maria, Brazil; ^5^Biomedical, Open University Foundation for the Third Age (FUnATI), Manaus, Brazil; ^6^Community University of Chapecó Region, Chapecó, Brazil; ^7^Federal University of Santa Catarina, Santa Catarina, Brazil; ^8^Department of Zootechnics and Biological Sciences, Federal University of Santa Maria, Santa Maria, Brazil; ^9^Open University Foundation for the Third Age (FUnATI), Manaus, Brazil; ^10^Department of Pathology, Federal University of Santa Maria, Santa Maria, Brazil

## Abstract

Diabetes mellitus is associated with chronic wound-healing problems that significantly impact patients' quality of life and substantially increase expenditure on healthcare. Therefore, the identification of compounds that can aid healing is justified. *Anredera cordifolia* (Ten.) has been used in folk medicine for curative purposes; however, the causal mechanisms underlying its healing effects remain to be elucidated. In this study, the effect of the ethanolic extract of *A. cordifolia* was evaluated in an *in vitro* healing model using fibroblasts cultivated under normoglycemic and hyperglycemic environments. The extract was predominantly composed of phytol and exhibited genoprotective activity. Fibroblast migration attenuated the adverse effects of hyperglycemia, favoring cell proliferation. Collagen levels were significantly increased in ruptured fibroblasts under both standard and hyperglycemic environments. The phytogenomic effect of the extract on three genes related to extracellular matrix formation, maintenance, and degradation showed that *A. cordifolia* increased the expression of genes related to matrix synthesis and maintenance in both normoglycemic and hyperglycemic individuals. Furthermore, it reduced the expression of genes related to matrix degradation. Overall, this is the first study to demonstrate the effectiveness of *A. cordifolia* in wound healing, elucidating possible causal mechanisms that appear to be based on the genoprotective effect of this plant on the migratory and proliferative phases of the wound healing process; these effects are probably related to phytol, its main constituent.

## 1. Introduction

Diabetes mellitus is a chronic systemic condition with an estimated global prevalence of 10.5% in 20–79-year-old individuals [1]. Diabetes triggers several types of clinical complications, such as wound healing impairment, which is closely related to the oxidative stress state caused by persistent hyperglycemia [2]. Considering the negative impact of diabetes mellitus on wound healing, many studies have sought to identify herbal medicines that could help treat this clinical condition [3, 4, 5].

*Anredera cordifolia* (Ten.) (Steenis) is a fleshy vine species belonging to the *Basellaceae* family with a natural distribution in South America. In Brazil, it is popularly called “fat leaf.” It is mainly cultivated by family farmers who preserve popular knowledge about its cultivation and consumption for medicinal purposes in topical skin lesions [6]. *A. cordifolia*, commonly known as binahong, has also been introduced in other regions of the world, such as Africa, Europe, and North America. *A. cordifolia* leaves are edible in both raw and cooked forms, with an excellent flavor and high protein value; notably, some of its biological properties have been reported in the literature [6]. The healing effect of *A. cordifolia* is potentially related to its chemical matrix, which contains compounds, such as saponins, tannins, alkaloids, diterpenoids, triterpenoids, flavonoids, phenolics, steroids, and glycosides found in extracts obtained from organic solvents because it is rich in lipids [7, 8, 9].

In Brazil, communities of Germanic origin use this plant to heal wounds. The collected leaves are washed or heated on a wood-burning stove, secured, and kept in contact with the wound using a clean cloth. This process is repeated for 1–3 days until the wounds heal. Experimental studies have confirmed the wound healing action of *A. cordifolia* [10, 11]. Complementary studies using rats fed a regular or high-fat diet for 6 weeks showed that *A. cordifolia* leaf extract could decrease blood glucose levels by regulating fat metabolism [12]. Furthermore, in a study on goat eye lenses exposed to glucose, *A. cordifolia* extract was found to exhibit antioxidant and anticataract properties [13].

Collectively, these results suggest that *A. cordifolia* can significantly affect wound healing in patients with diabetes. To test this hypothesis, the present study aimed to evaluate the effect of an ethanolic extract of *A. cordifolia* on the healing process of skin lesions *in vitro* using commercial human cells (HFF-1 fibroblasts) cultivated under normoglycemic and hyperglycemic environments. The potential mechanism of action of *A. cordifolia* in the context of antioxidant and collagen metabolism was also investigated in this study.

The relevance of this topic is justified by the fact that, despite its use by populations, no scientific studies have been found that evaluate the causal mechanisms related to the action of *A. cordifolia* in hyperglycemic/diabetic environments.

## 2. Materials and Methods

### 2.1. Reagents and Equipment

The following equipment was used to obtain the extract and perform chemical characterization: a rotary evaporator (IKA brand—Model RAV 10), an Agilent GC-7890B chromatograph coupled to a quadrupole mass spectrometer (5977A) (Agilent Technologies, Palo Alto, CA, USA). All analyses involving the measurement of absorbance or fluorescence were performed using a SpectraMax i3x multimode microplate reader (Molecular Devices, San Jose, USA). All flow cytometry analyses cells were performed on a BD Accuri™ C6 Plus (BD Pharmingen™; San Jose, CA, USA). Gene expression was determined using qRT-PCR on a Rotor-Gene Q 5plex HRM System (Qiagen, Hilden, Germany). RNA was quantified using a Thermo Scientific NanoDrop™ 1.000 Spectrophotometer. Histomorphological analyses were performed using a Leica 107 DMI 4000B microscope (Leica Microsystems GmbH, Wetzlar, Germany). The reagents and plastics used in the experiments were purchased from Supelco Co., Invitrogen Co., and Sigma–Aldrich Co. (St. Louis, MO, USA). The panoptic dye kit was purchased from Panzootic Dye—Laborclin® (Belo Horizonte, MG, Brazil). Molecular reagents including the Quant-iT™ dye PicoGreen® dsDNA kit were purchased from Invitrogen, Life Technologies (Carlsbad, CA, USA), Bio-Rad Laboratories, Hercules, CA-USA, and Biotechnology Co. (Alvorada, RS, Brazil). The QuantiFast SYBR Green PCR Kit was purchased from Qiagen (Germany).

### 2.2. General In Vitro Experimental Design

**Figure 1:**
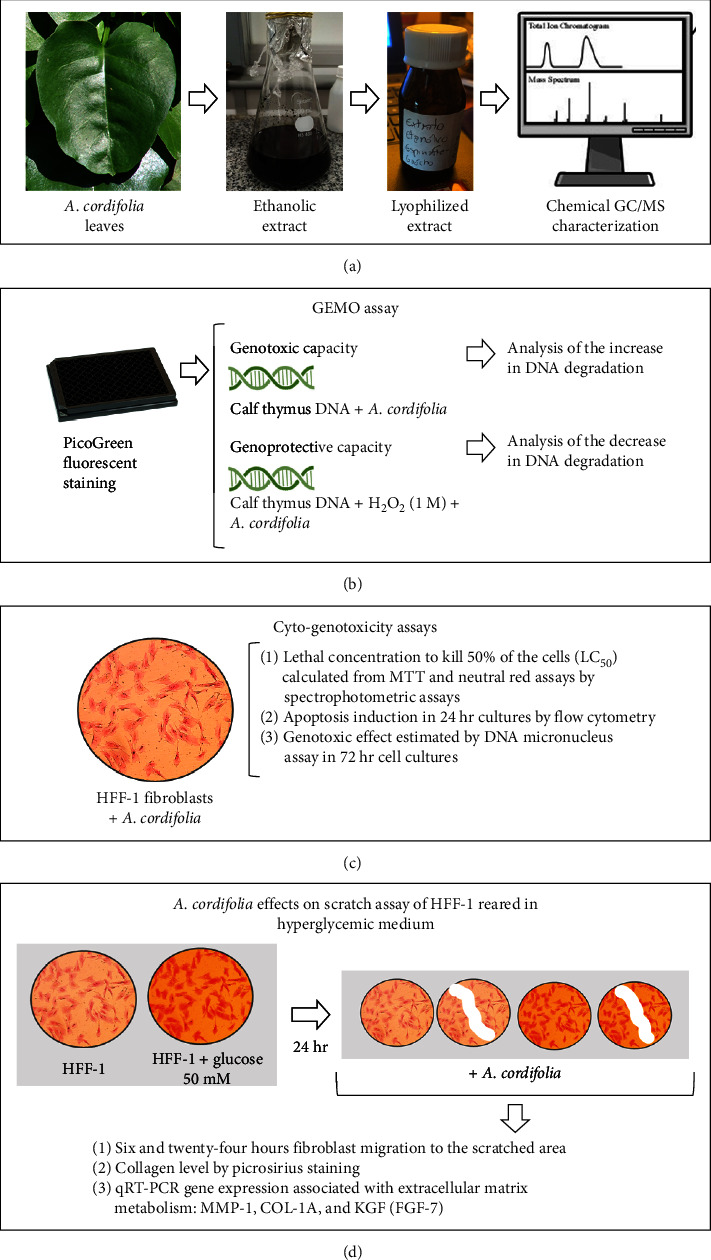
(a–d) General experimental design and main laboratory protocols used in the study.

This study was designed in four stages, as shown in Figure 1. In the first stage, after obtaining the leaves of *A. cordifolia*, ethanolic extraction and chemical characterization were performed [13]. Considering that many plants may possess desirable biological properties but may also have adverse effects, the second stage of the study evaluated whether the extract exerted any genotoxic impact by inducing mutagenesis or, conversely, possessed genoprotective capabilities by preventing the degradation of double-stranded DNA (dsDNA) molecules exposed to an oxidizing agent. This protocol was conducted because the plant and its extract are commonly used directly for treating open skin lesions. To this end, a noncellular test to evaluate genomodifying capacity (GEMO) was performed [14].

In the third stage, the concentration range at which the *A. cordifolia* extract did not exhibit cytotoxicity or genotoxicity in human dermal fibroblast cultures was determined [15]. Finally, the fourth stage of the study assessed the effect of *A. cordifolia* on scratched fibroblast monolayers cultured in a hyperglycemic medium that mimicked the physiology of diabetes mellitus *in vitro* [16]. At this stage, the effect of *A. cordifolia* extract was investigated in terms of (1) the rate of fibroblast migration to the scratched area, (2) the levels of collagen produced by the culture, and (3) the modulation of genes related to extracellular matrix production, which is vital for wound healing.

### 2.3. Preparation of the Plant Hydroalcoholic Extract

*A. cordifolia* leaves were collected in February 2022 (summer) from the municipality of Palmeira das Missões, RS, in the south of Brazil (27°55′19′S, 55°19′13′W), a humid subtropical region. The plant voucher specimen was deposited at the herbarium of the Federal University of Santa Maria, Palmeira das Missões (UFSM, number 1,514). In total, 480 g of leaves were collected manually, separated, and dried at room temperature (25°C) to a constant weight (~5 days), then packed in plastic bags, identified, and stored at 4°C until extraction. The following steps were performed to calculate the extract production yield: (1) weighing the plant leaves; (2) extraction using ethanol; (3) rotary evaporation and freeze-drying; (4) weighing the final extract, that is, the obtained powder. From 28.39 g of dried and ground *A. cordifolia* leaves, 0.63 g of lyophilized ethanol extract was obtained, resulting in a yield of 2.2%. This percentage was calculated as follows: Yield (%) = (Weight of the final extract/Starting weight of the leaves) ×100.

The *A. cordifolia* extract was subjected to chemical characterization in the Chemistry Laboratory at the Community University of Chapecó Region (UNOCHAPECO) (Figure 1(a)). Considering the procedures commonly employed by traditional communities regarding the application of *A. cordifolia* on wounds, where leaves are not boiled before use, this study involved a rapid ethanol extraction, evaluation of the primary bioactive molecules obtained, and their effect on fibroblasts. Partner laboratories obtained the extract following a protocol similar to that performed by Feriyani et al. [13], as follows: the dried plant matrix was ground using a mortar and pestle to reduce particle size and increase the contact surface area with 99% ethanol for analysis (P.A.), for an enhanced extraction yield. Dried and ground samples were obtained and placed in a 250 mL Erlenmeyer flask. Then, 250 mL of HPLC-grade ethanol was added as the solvent. After 48 hr, the saturated extraction solution was removed and stored and another 250 mL of ethanol was added to the sample in the Erlenmeyer flask to optimize the extraction. The saturated solution was removed, mixed with the previous solution, and filtered through a filter paper.

Ethanol was evaporated from the final mixture using a rotary evaporator at 40°C. The material remaining after rotary evaporation was lyophilized and used for chemical characterization and *in vitro* experiments.

### 2.4. Chemical Characterization of the *A. cordifolia* Leaves Ethanolic Extract

The chemical compounds in the ethanolic extract of *A. cordifolia* were detected using gas chromatography/mass spectrometry. For this analysis, 10 mg of the extract was diluted in 10 mL of methanol and this sample was injected into the chromatograph at a volume of 1 mL. Chromatography began at 150°C with a temperature increase rate of 12°C/min until it reached 280°C. The rate was then increased at 20°C/min until the temperature reached 300°C and was kept constant for 10 min. An Agilent 19091s-433:93.92873 HP-5MS 5% phenyl methyl silox column with dimensions 30 m × 250 *µ*m × 0.25 *µ*m was employed. Helium was used as the carrier gas at a flow rate of 1.2 mL/min. For GC–MS detection, an electron ionization system was used with an ionization energy set at 70 eV and a mass range of *m*/*z* 40–350. The chemical components were detected and identified by comparing the mass spectra using the NIST 5.01 Mass Spectral Library (Agilent P/N G1033A). The relative quantity of each element was calculated based on its respective peak in the chromatogram.

### 2.5. Genomodifier Capacity (GEMO) Assay

The GEMO assay was performed as described by Cadoná et al. [14] (Figure 1(b)) based on the increased breakage rate of double-stranded DNA (dsDNA) molecules upon exposure to H_2_O_2_ at 3 M for 30 min. The dsDNA breakage is observed through exposure to the fluorescent dye DNA PicoGreen®, which has a high affinity for dsDNA but not for single-stranded DNA molecules or nucleotides. In this context, when a break occurs, the fluorescence emitted by the binding of dsDNA to the dye is decreased. The GEMO assay was performed using a 96-well dark plate, with wells containing 10 *μ*L of dsDNA (1 mg/mL), 100 *μ*L of *A. cordifolia* extract plus 90 *μ*L of TE buffer to obtain a final volume of 200 *μ*L. After 30 min, PicoGreen® fluorescent dye (TE buffer 1 : 200) was added to the wells, and fluorescence was observed after 5 min at 480 nm for excitation and 520 nm for emission. Fluorescence is compared with the control group (dsDNA only) and genotoxicity is identified by the decrease in fluorescence. To evaluate the genoprotective capacity, 10 *μ*L of dsDNA (1 mg/mL) were incubated with 100 *μ*L of H_2_O_2_ (3 M), the pro-oxidant, and 100 *μ*L of *A. cordifolia* extract at different concentrations. After 30 min of incubation, PicoGreen® fluorescent dye (TE buffer 1 : 200) was added. After 5 min of incubation, fluorescence was observed at 480 nm for excitation and 520 nm for emission.

### 2.6. Fibroblast Cell Culture Conditions

Fibroblasts of the HFF-1 lineage (SCRC-1041™) manufactured by the American Type Culture Collection (ATCC®-USA) were purchased by the Rio de Janeiro Cell Bank. HFF-1 cells are derived from human foreskin tissue and have been used in studies related to skin biology, wound healing, tissue engineering, and aging [17, 18].

Cells were cultured in low-glucose Dulbecco's modified Eagle's medium (DMEM) (1 g/L) supplemented with 15% fetal bovine serum, 1% antibiotics (penicillin/streptomycin), and 1% antifungal (Amphotericin B). To evaluate the effects in standard and high-glucose environments, some cells were grown in a culture medium supplemented with 50 mM glucose, to mimic a high-glucose state. Cells were cultured according to the protocol described by Filho [19].

### 2.7. Cytotoxicity Assays

The potential cytotoxic effects of *A. cordifolia* extract were assessed using three complementary protocols (Figure 1(c)). The first protocol evaluated cell viability using a 3-[4,5-dimethylthiazol-2-yl]-2,5-diphenyltetrazolic bromide (MTT) spectrophotometric assay in 24-hr fibroblast cultures [20]. The results were used to calculate the lethal concentration required to kill 50% of the cells (LC50). The second protocol confirmed the potential cytotoxic effects of *A. cordifolia* through cell viability analysis using propidium iodide (PI) staining and flow cytometry. Additional flow cytometry analysis was performed to identify whether exposure to *A. cordifolia* extract could induce and increase fibroblast apoptosis. Cytotoxicity was evaluated using an MTT assay in 24 hr cell cultures exposed to *A. cordifolia* extract at 0.1, 0.3, 1.0, 3.0, and 10 *µ*g/mL concentrations. The culture supernatant was removed, and the cells were resuspended in phosphate-buffered saline (PBS; 0.01 M, pH 7.4), and MTT reagent dissolved in 5 mg/mL PBS was added to a 96-well plate containing the sample treatments and 5 × 10^5^ cells per well. The reaction was then incubated for 1 hr at 37°C according to the Assay Guidance Manual published by Riss et al. [21]; laboratory analyses (data not shown) showing similarities between the results of 1 and 4 hr were transformed into percentage of the control and compared with each other. A similar MTT assay protocol was previously described by Filho [19], using HFF-1 cell cultures. After incubation, the supernatant was removed and discarded, the cells were resuspended in 200 *µ*L DMSO, and absorbance was measured at 570 nm.

Flow cytometry was used to determine apoptotic events using Annexin-V-FITC and PI staining kits, according to the manufacturer's instructions. Cells were seeded into six-well plates at 1 × 10^6^ cells/well in 2 mL of DMEM containing different *A. cordifolia* extract concentrations, and incubated for 24 hr. After incubation, the cells were trypsinized and resuspended in 1x Binding Buffer at 1 × 10^6^ cells/mL. Then, 100 *µ*L of the cell suspension was transferred to a microtube and then mixed with 5 *µ*L of Annexin V FITC and 5 *µ*L of PI. After gently vortexing the cells, they were incubated for 15 min at room temperature (25°C) in the dark. Subsequently, 400 *µ*L of 1x binding buffer was added to each tube, and flow cytometry analysis was performed according to their forward scatter/side scatter (FSC/SSC) patterns. Data acquisition and cell content analyses were performed using FlowJo vX.0.7 software (Tree Star, Inc., Ashland, OR, USA).

### 2.8. DNA Micronucleus Assay

An additional safety test for the exposure of fibroblasts to *A. cordifolia* extract was performed by analyzing irreversible DNA damage assessed by the DNA micronucleus assay, as previously described by Da Cruz et al. [18], with slight modifications to the protocol described by the Organization for Economic Co-operation and Development [22]. Briefly, fibroblasts (1 × 10^3^ cells) were transferred to 24-well plates. After 24 hr, the culture medium was replaced with medium containing *A. cordifolia* extract at different concentrations. The fibroblasts were then maintained for 72 hr to obtain a greater number of mitotic cells, which enabled the detection of micronuclei and other nuclear alterations. From fibroblast monolayer cultures, the medium was gently removed using a Pasteur pipette, the cells were washed with PBS, and stained with 4′,6-diamidino-2-phenylindole (DAPI), previously prepared by dissolving 14.3 mM dihydrochloride in 2 mL of deionized water with 0.5% of DMSO. As DAPI can access and bind to DNA molecules to produce bright blue fluorescence, it is possible to identify the nucleus and its potential alterations in the cells. After staining, the plates were analyzed under a fluorescence optical microscope with excitation between 455–461 nm. In addition to micronuclei (MN) identification, DAPI staining allowed identification of the following nuclear alterations: nuclear nondisjunction (NDi), nuclear dimorphism (NuD), and nuclear apoptosis. Microscopic analysis was performed at 20x magnification of fluorescence using ImageJ software to amplify the images and perform quantitative measurements, including the nuclear perimeter. The frequency of MN and other nuclear alterations was scored based on the analysis of 1,000 cells per treatment. The images were inverted to produce white nuclei and facilitate the identification of MN and other nuclear alterations.

### 2.9. Collagen Quantification by Picrosirius Staining

The effect of *A. cordifolia* extract on fibroblast collagen metabolism was evaluated using picrosirius red staining that relies on the birefringence of collagen fibers, which can then be detected by microscopy or spectrophotometric analyses [23]. Therefore, the collagen concentration was measured by spectrophotometry using picrosirius red dye in fibroblast cultures after 72 hr of treatment, as described by Da Cruz et al. [18]. This protocol involved degradation of the monolayer culture with a trypsin solution and moderate mechanical agitation. After centrifugation (1,500 rpm for 10 min), the supernatant was discarded to remove potential collagen residues that could have remained on the outer membrane of the cells, after the addition of PBS, they were centrifuged again at 1,500 rpm for 10 min.

Then, 250 *µ*L/well of 0.1% Sirius Red Stain dissolved in saturated picric acid was added to each well, and the 96-plate was incubated for 1 hr at room temperature.

The dye solution was then removed using a pipette, and the cells were incubated for 30 s in a solution containing 150 *μ*L of 0.01 M hydrochloric acid to remove the excess dye that did not bind to collagen molecules. The wells were then incubated with 150 *μ*L of 0.1 M NaOH for 30 min at room temperature. Afterward, 100 *μ*L aliquots of the solutions in the wells were transferred to a new 96-well plate and absorbance was measured at 492 nm.

### 2.10. Fibroblast Scratch Assay

An *in vitro* assay was conducted to assess the effect of *A. cordifolia* extract on the migration and proliferation of fibroblasts cultured in hyperglycemic medium using the scratch assay, as previously described by Nicolau et al. [16] with slight modifications (Figure 1(d)). HFF-1 cultures were initially exposed to a hyperglycemic medium with 50 mM glucose for 48 hr. The cultures were then exposed to *A. cordifolia* extract and subjected to the scratch protocol. To conduct this assay, culture plates were precoated with 400 *µ*L of 50 *µ*g/mL poly-L-lysine and incubated for 2 hr at 37°C before adding the cells. On the following day, a *Scratch Assay* was performed (by creating a vertical scratch with a 200 *µ*L (yellow) pipette tip on the adhered cells at the bottom of the well), followed by treatment with 1 *µ*g/mL of *A. cordifolia* extract. To enhance the visualization of fibroblasts, the cultures were fixed and subjected to panoptic fast staining, as described by Da Cruz et al. [18]. Briefly, the culture plates were centrifuged for 2 min at 1,000× *g* with supernatant removal and the addition of methanol-based fixative solution (100 *µ*L) for 5 min. Next, the fixative solution was removed, and 100 *µ*L of eosin dye was added for 2 min after exposure to a third blue solution (composed of methylene blue and deionized water). Excess dye solution was then removed, and the wells containing the cells were air-dried at room temperature. The fibroblast cytoplasm and its processes were stained light pink, whereas the nuclei were stained more strongly with a purplish-pink color. After 6 and 24 hr, the migration rate of fibroblasts to the area of the scratch was assessed by counting the cells present in the cell-free area and measuring the distance (mm^2^) between the cells on each side of the scratch using an optical microscope at 10x magnification.

### 2.11. Gene Expression by qRT-PCR Assay

The potential phytogenomic role of *A. cordifolia* in modulating the gene expression of critical molecules for wound healing was evaluated considering that alterations in this process can lead to the formation of hypertrophic keloids or failure of wound closure, which is a common clinical condition in diabetic patients. Therefore, the effect of *A. cordifolia* extract on the expression of three genes involved in the wound healing process was investigated in scratched fibroblasts in 24-hr cultures. The functional relevance of each gene and the primers used in the qRT-PCR assays are listed in Table 1.

Transcript quantification of each gene was performed using total RNA samples extracted with TRIzol, following the manufacturer's instructions, and quantified at a wavelength of 532 nm. For reverse transcription, RNA samples (1 *μ*g/mL) were treated with 0.2 *μ*L of DNase at 37°C for 5 min, followed by heating at 65°C for 10 min. The cDNA was generated using 1 *μ*L of iScript cDNA and 4 *μ*L of iScript Mix in a reaction consisting of the following steps: heating at 25°C for 5 min, at 42°C for 30 min, and at 85°C for 5 min, followed by incubation at 5°C for 60 min. qRT-PCR was performed in a 20 *μ*L reaction volume with 1 *μ*L of cDNA, 1.3 *μ*L of each primer, and 16.4 *μ*L of QuantiFast SYBR Green PCR mix. The following qRT-PCR parameters were used: 95°C for 3 min, followed by 40 cycles of 95°C for 10 s, 60°C for 30 s, and a melt curve at 65°C. *β*-Actin was used as the housekeeping gene, and relative expression was calculated using the comparative Ct method, expressed as the fold expression compared to the control.

### 2.12. Statistical Analysis

Data were analyzed using GraphPad Prism v. 9.4.0. The protocols followed in this study are based on promising *in vitro* practices recommended by the Organization for Economic Co-operation and Development Guidance Document on Good *In Vitro* Method Practices (OECD, 2018). The data from the cell culture protocols were transformed into percentages relative to the control because *in vitro* protocols can increase the variance caused by experimental errors except for the following variables: cell cycle phases (%), micronuclei and other nuclear alterations (%), fibroblast migration, number of grids with cells or cytoplasmic prolongations, and gene expression values were evaluated by mean ± standard deviation (SD) (mean ± SD). Assays involving three or more treatments were compared using a one-way analysis of variance (ANOVA) followed by the Tukey's post hoc test). The frequency of nuclear alterations, including MN, was compared using chi-square tests, and the results are presented as *n*/1,000 nuclei. Statistical significance was set at *p* < 0.05.

## 3. Results

### 3.1. The Ethanolic Extract of *A. cordifolia* Is Rich in Phytol and Presents Genoprotective Capacity

**Figure 2:**
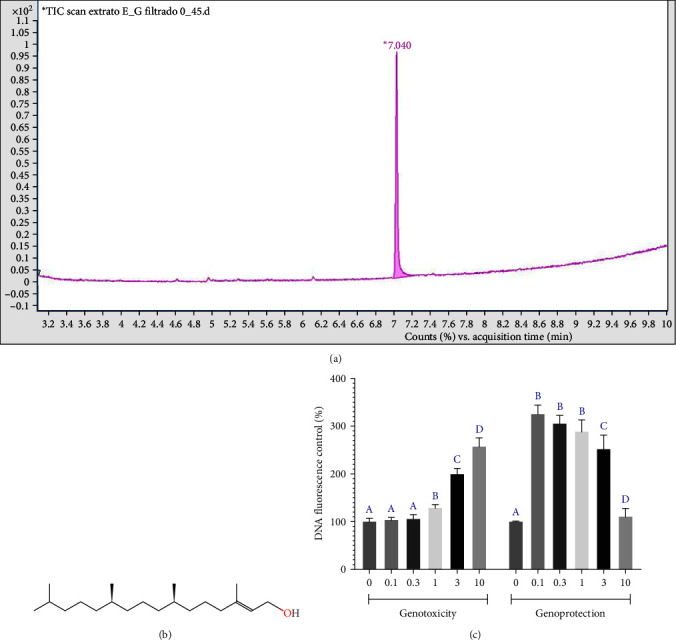
Chemical characterization of *A. cordifolia* leaf ethanolic extract and genomodifier capacity (GEMO) assay data: (a) chromatogram; (b) chemical molecule structure; and (c) dsDNA intensity (percentage of control) of different *A. cordifolia* concentrations on pure DNA molecules, measured using PicoGreen dye. In the analysis of genotoxic capacity, a decrease in the dsDNA fluorescence rate indicates degradation of the molecule by the extract. In the analysis of genoprotective capacity, an increase in the fluorescence rate indicates protection of dsDNA exposed to H_2_O_2_ (3 M), which causes extensive breakdown of dsDNA molecules. This genoprotective effect was measured to determine the potential effect reverses the rate of dsDNA degradation induced by exposure to H_2_O_2_. As there is a natural rate of dsDNA degradation, the increase in fluorescence observed in the part of the graph that shows the genotoxicity data indicating that the *A. cordifolia* extract delayed the natural process of dsDNA degradation, as the fluorescence increased. A statistical comparison among *A. cordifolia* concentrations was performed using a two-way analysis of variance followed by a Tukey's post hoc test. Different letters in each assay indicate statistical significance at *p*  < 0.05.

Six peaks were observed in the GC–MS chromatogram of *A. cordifolia* extract. However, ≥95% area peak was identified as phytol (molecular weight = 296.5310, chemical formula = C_20_H_40_O; count × acquisition time = 7.04, retention time = 20–25 min). Phytol has the following synonyms: (1) (2-hexadecen-1-ol, 3,7,11,15-tetramethyl, (R- (R+,R+- (E))]; (2) trans-phytol; (3) 3,7,11-15-tetramethyl-2-hexacen-1-ol, (2E, 7R,11R); (4) (23,75,115)-3,7,11,15-tetramethyl-2-hexacen-1-ol; and (5) 3,7,11,15-tetramethyl 2-hexacene-1-ol, (23,75,11, R) (Figures 2(a) and 2(b)).

A reduction in the dsDNA fluorescence rate was anticipated when assessing genotoxic potential (GEMO assay), suggesting degradation of the molecule by the extract. When evaluating the genoprotective potential, an increase in the fluorescence rate was observed, signifying the safeguarding of dsDNA exposed to H_2_O_2_ (3 M), which induces extensive degradation of dsDNA molecules (Figure 2(c)).

### 3.2. Cytotoxicity of the Alcoholic Extract of *A. cordifolia* in Dermal Fibroblasts

**Figure 3:**
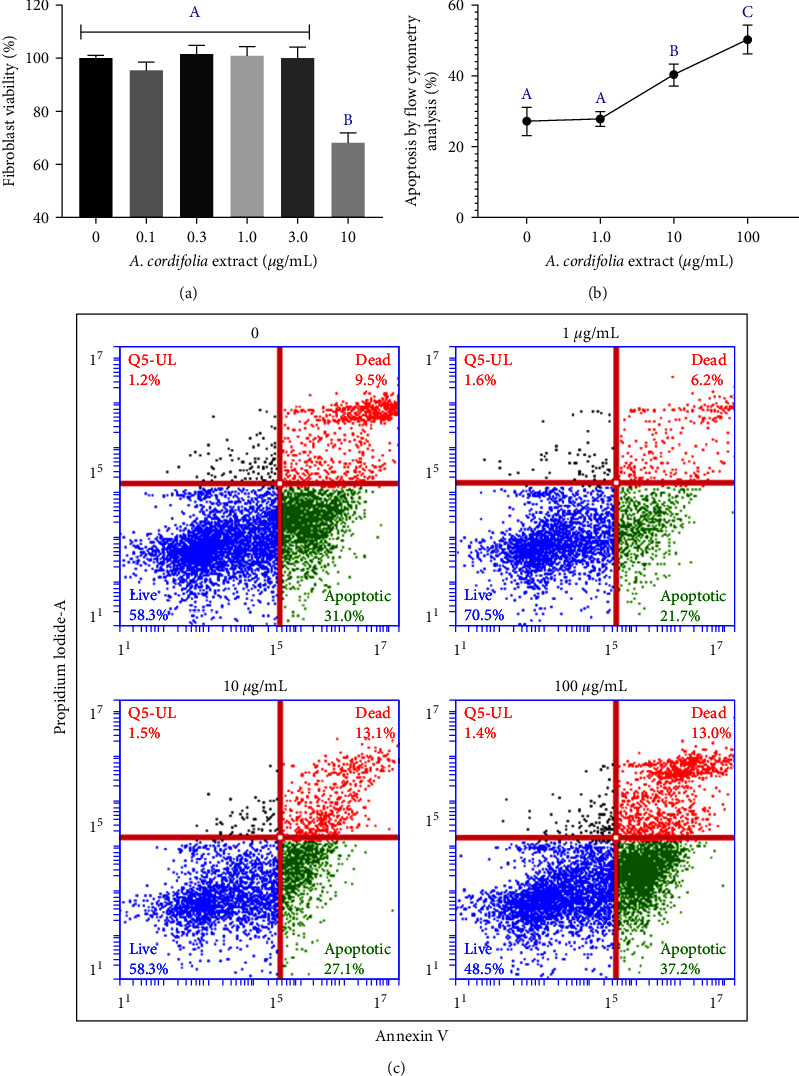
Cytotoxic evaluation of *A. cordifolia* ethanolic extract in 24-hr-exposed HFF-1 dermal fibroblast cultures: (a) MTT viability assay; (b) comparison of the percentage of apoptotic and already apoptotic cell death based on flow cytometry analysis between the controls and fibroblasts exposed to 1, 10, and 100 *µ*g/mL of *A. cordifolia* extract. (c) Flow cytometry-based graphical representation with PI and Annexin V used for the detection of apoptotic events. The cytotoxic effect of different concentrations of *A. cordifolia* was compared using a one-way analysis of variance (ANOVA) followed by a Tukey's post hoc test. Other letters (A, B, and C) indicate significance in the post hoc test with *p*  < 0.05.

Based on the GEMO assay, we increased the concentration of *A. cordifolia* by a factor of 10 in the viability tests to simulate its LC_50_. From the MTT viability assay of fibroblasts incubated for 24 hr with *A. cordifolia* extract, an LC_50_ = 113.81 (95%CI = 99.11–224.3) *µ*g/mL was obtained. The neutral red assay confirmed that 10 *µ*g/mL extract significantly decreased the fibroblast viability compared with that in the controls (*p* ≤ 0.01) (Figure 3(a)). A complementary analysis was performed to evaluate whether *A. cordifolia* could induce apoptosis. As shown in Figure 3(b) and representative flow cytometry charts (Figure 3(c)), from 10 *µ*g/mL concentration, *A. cordifolia* significantly increased the apoptotic events detected using the flow cytometry Annexin V-PI protocol in comparison to that in controls (*p* ≤ 0.001).

### 3.3. Ethanolic Extract of *A. cordifolia* Has Low Genotoxicity in Dermal Fibroblasts

**Figure 4:**
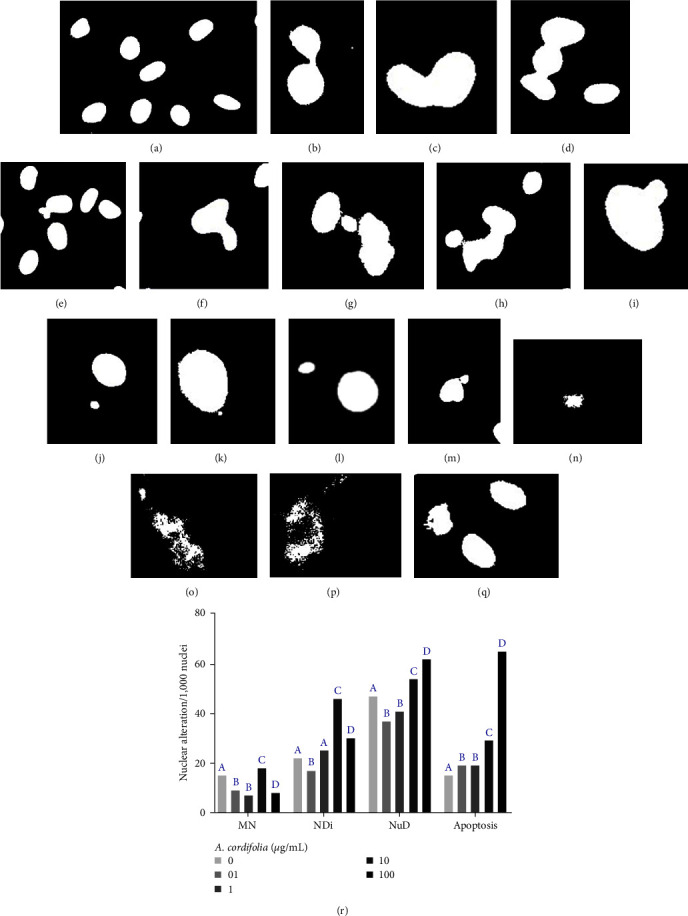
Effect of *A. cordifolia* alcoholic extract at different concentrations on the rate of micronucleus (MN) formation and other nuclear alterations (NDi = nuclear dimorphism; NuD = nuclear disjunction, and nuclear apoptosis) using DAPI fluorescent staining, wherein the fluorescent blue staining was altered to white staining for facilitating the identification of nuclear alterations. (a) Microphotographs representing healthy nuclei; (b–q) representative MN, and other nuclear alterations; (b–d) NDi examples; (e–h) NuD examples; (i–m) MN examples; (n–q) nuclear apoptosis examples. (r) The frequency of MN and other nuclear alterations among the control and 72-hour fibroblast cultures exposed to *A. cordifolia* extract at different concentrations. In each treatment, 1,000 nuclei were analyzed using Image J software. A comparison of the absolute frequencies of MN and other nuclear changes was performed using the *χ*^2^ test, with different letters indicating statistically significant differences at *p* < 0.05 in each nuclear alteration category.

The potential genotoxic effects of *A. cordifolia* through nuclear alterations, particularly the formation of MN, were analyzed and the key findings are summarized in Figure 4. Microscopic analysis revealed several alterations including Ndi (Figures 4(b), 4(c), 4(d), and 4(e)), NuD (Figures 4(f), 4(g), and 4(h)), MN (Figures 4(i), 4(j), 4(k), 4(l), 4(m), and 4(n)), and nuclei undergoing apoptosis (Figures 4(o), 4(p), 4(q), and 4(r)).

Comparison of the frequency of these alterations (Figure 4(r)) revealed a decrease in the MN rate in fibroblasts exposed to concentrations of 0.1 and 1 *µ*g/mL of the *A. cordifolia* alcoholic extract. However, a significant increase in MN occurred at the 10 *µ*g/mL concentration. Surprisingly, the 100 *µ*g/mL concentration reduced the MN frequency compared to that in the control fibroblasts. The NDi frequency was lower in cultures exposed to 0.1 *µ*g/mL and similar to the control in those exposed to 1 *µ*g/mL of the extract. In contrast, the NI rate was higher in fibroblasts exposed to higher concentrations of the *A. cordifolia* extract. The occurrence of NuD was also lower in cultures exposed to lower concentrations of *A. cordifolia*, while concentrations of 10 and 100 *µ*g/mL induced a higher number of cells with nuclear morphological alterations. A significant increase in the number of apoptotic nuclei was observed in cultures exposed to the *A. cordifolia* extract compared with those in the control. However, this result was more pronounced in plants exposed to the highest concentrations tested.

Based on the results obtained in the initial assays, remaining protocols were performed using a concentration of 1 *µ*g/mL, as it represents one of the lowest concentrations with potential beneficial effects as well as safety, considering the potential cyto-genotoxicity of the alcoholic extract of *A. cordifolia*.

The *A. cordifolia* extract attenuates the adverse effects of hyperglycemia in scratched fibroblasts, increasing the collagen concentration in the supernatant.

**Figure 5:**
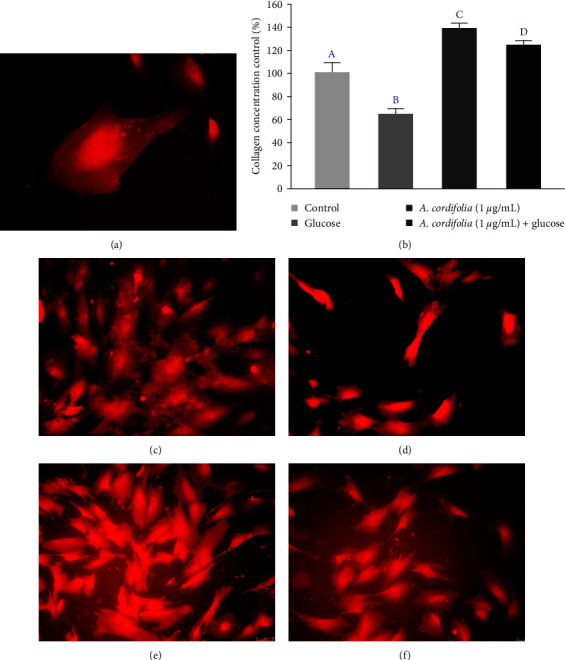
Fibroblast migration from the scratch assay after 6 and 24 hr in normoglycemic and hyperglycemic cultures exposed to *A. cordifolia* alcoholic extract. The migration rate was assessed by the presence of fibroblasts or their cytoplasmic extensions in microphotographs divided into 99 grids, each with an area of 173.47 × 130.10 mm^2^. The central region represents the site where the monolayer was scratched with the tip of a pipette. Fibroblasts were stained with Panoptic for better visualization and analyzed at 200x magnification. (a) Representative microphotographs from 6 hr after the fibroblast monolayers were scratched. The arrow indicates the average area of the injury represented by 5–6 grids with a total estimated area of 867.35–1,040.82 × 650.5–780.6 mm^2^; (b) representative microphotographs from 24 hr after the fibroblast monolayers were scratched; (c) comparison among treatments of the mean ± SD of grids that had fibroblasts or their extensions indicating migration after 6 hr in which the central region was scratched. (d) Comparison among treatments of the mean number ± SD of grids with fibroblasts or their extensions indicating migration after 24 hr in which the central region was scratched. Both comparisons were conducted using ANOVA followed by Tukey's post hoc analysis. (e) Representative microphotography from fibroblasts exposed to *A. cordifolia* extract. (f) Representative microphotography from fibroblasts reared in hyperglycaemic medium plus *A. cordifolia* extract. Statistical differences were identified by different letters with *p*  < 0.05.

The concentration of 1 *µ*g/mL *A. cordifolia* extract was chosen for this protocol based on previous results that indicated its genoprotective and noncytotoxic effects. The effect of *A. cordifolia* extract on fibroblast cultures exposed to glucose was evaluated 6 and 24 hr after the monolayers were scratched. The main results are shown in Figure 5. Fibroblast migration after 6 hr (Figures 5(a) and 5(c)) of the injury was similar between the control and cultures exposed to *A. cordifolia* extract. In contrast, fibroblasts cultured in the hyperglycemic medium exhibited delayed migration, as observed by the occupation of cells and/or their extensions in grids located at the scratch site. In the presence of *A. cordifolia*, the migration rate of glucose-exposed fibroblasts was similar to that of controls, indicating that the extract attenuated the negative effects of hyperglycemia.

In 24-hr cultures, fibroblasts exhibited a lower migration rate in glucose-exposed cultures than in the controls (Figure 5(b)). In contrast, exposure to *A. cordifolia* extract resulted in significantly higher migration than that of the control. In hyperglycemic medium, exposure to *A. cordifolia* extract reversed the sluggishness of the migration rate of fibroblasts, making it similar to that of the controls.

**Figure 6:**
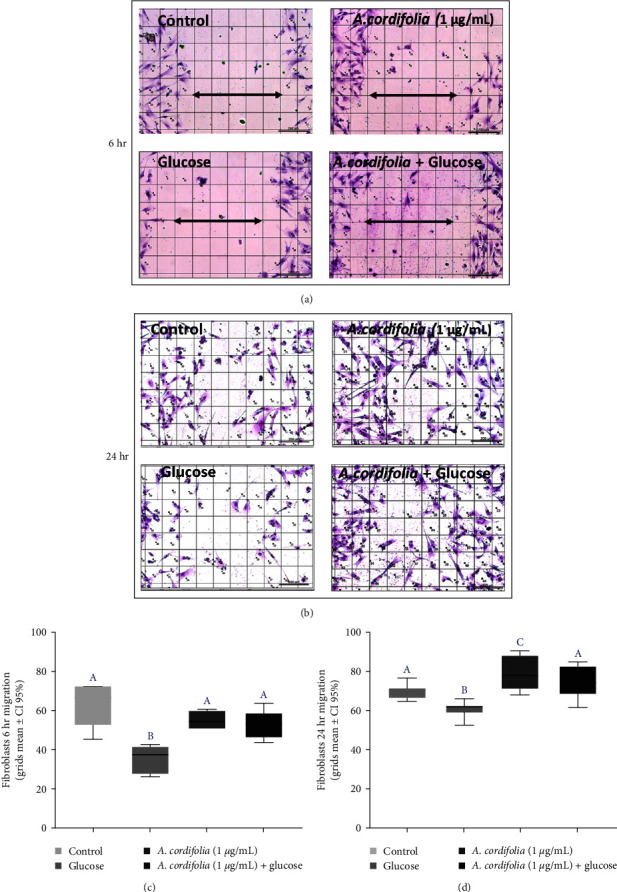
Effect of the alcoholic extract of *A. cordifolia* on collagen production from the dermal fibroblast monolayer scratched cultures grown in the normoglycemic and hyperglycemic medium for 72 hr. Collagen quantification was performed by spectrophotometry using Picrosirius staining. Confirmation of collagen production was conducted using the same picrosirius dye in monolayer cultures analyzed by fluorescence microscopy (×200 magnification). (a) Representative fibroblast cells showing intracellular and extracellular collagen deposition. (b) Comparison of collagen production among different treatments analyzed by ANOVA followed by Tukey's post hoc test considering significant all values with *p*  < 0.05. Statistical differences are identified by different letters. (c) Representative microphotographs from control fibroblasts. (d) Representative microphotographs from fibroblasts cultured in hyperglycemic medium showing retention in the collagen molecules in cells.

This protocol also assessed the effect of *A. cordifolia* on collagen production (Figure 6); a significant increase in the levels of collagen was observed in torn fibroblasts exposed to the plant extract compared to that in the controls. In a hyperglycemic environment, the collagen concentration decreased, but this outcome was reversed when torn fibroblasts were simultaneously exposed to the *A. cordifolia* extract.

### 3.4. Cordifolia Extract Differentially Modulates Gene Expression Related to Extracellular Matrix Formation in Scratched Fibroblasts

**Figure 7:**
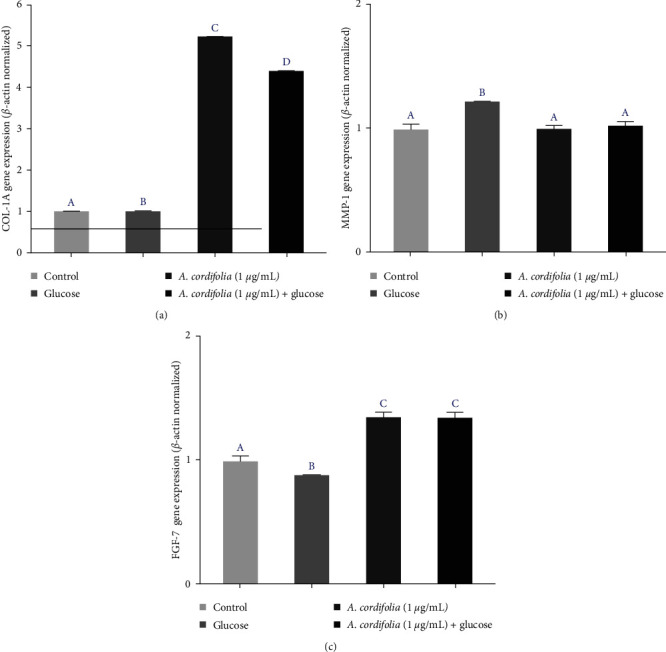
Phytogenomic effect on the modulation of gene expression involved in the metabolism (formation, maintenance, and degradation) of extracellular matrix by the alcoholic extract of *A. cordifolia* in scratched fibroblasts cultured in normoglycemic and hyperglycemic media. Results are normalized to *β*-actin gene expression and compared with the controls gene expression indicated as 1: (a) COL-1A gene expression; (b) MMP-1 gene expression; and (c) FGF-7 gene expression. Differences in gene expression were compared using one-way ANOVA followed by Tukey's post hoc test. Different letters indicate significant differences between the treatments with *p*  < 0.05.

The phytogenomic effects of *A. cordifolia* extract on three genes related to extracellular matrix metabolism were investigated in scratched fibroblast monolayers cultured with and without high glucose concentrations. In fibroblasts cultured under hyperglycemic conditions, the expression of COL-1A and FGF-7 genes was downregulated compared with that in the control. However, exposure to the *A. cordifolia* extract induced COL-1 overexpression in normoglycemic and hyperglycemic cultures (Figures 7(a) and 7(c)). Regarding the MMP-1 gene, glucose-induced overexpression, while exposure to *A. cordifolia* did not modulate this gene differently than in the control group under both normoglycemic and hyperglycemic cultures (Figure 7(b)).

## 4. Discussion

Several plants have been used in traditional medicine. About 20% of the plants found in the world have had their effects demonstrated through pharmacological or biological tests [24, 25]. For example, *Calotropis gigantea* and *Calotropis procera*, traditionally used by populations in Indonesia, Malaysia, China, and India as antifungal, antipyretic, and analgesic agents, have had their potential antimicrobial effects proven through the studies by Kar et al. [24] and Pattnaik et al. [26].

However, studies of biological properties and safety remain in the early stages. This is the case with *A. cordifolia*, which is popularly employed as a phytotherapeutic agent in healing skin wounds and infections. This study evaluated the potential of an alcoholic extract of *A. cordifolia* to modulate specific wound healing-related mechanisms in normoglycemic and hyperglycemic culture media. Surprisingly, the primary bioactive molecule in the alcoholic extracts was phytol. The phytol-rich extract exhibited no genotoxicity at low concentrations and induced significant fibroblast migration in scratched monocultures. The *A. cordifolia* extract also modulated extracellular collagen levels and exerted phytogenomic properties by modulating COL-1A and FGF-7 gene expression. Furthermore, the results of this study suggest that *A. cordifolia* extract might be especially beneficial in patients with diabetes who tend to experience hyperglycemic states that adversely affect wound healing processes. The interpretation of these results is discussed in detail below.

The initial findings were related to the chemical matrix of *A. cordifolia* leaves, particularly those involving alcoholic extraction. The various bioactive molecules extracted from the leaves of this species depend on the solvent and extraction time. Primary studies related to the characterization of the chemical matrix of *A. cordifolia* are summarized in Table 2. Previous studies have also identified phytol in the leaf extracts of *A. cordifolia* [9, 34] and as a volatile constituent of extracts the from the aerial parts (leaves and stems) of this plant [40]. However, variations in the components of the chemical matrix *A. cordifolia* were evaluated by Dwitiyanti et al. [38], who attempted to standardize the extraction conditions to obtain extracts with a consistent chemical profile and biological activity. The authors obtained and chemically characterized distinct types of aqueous and alcoholic extracts from *A. cordifolia* leaves and observed significant variations. Based on these results, this plant can be inferred to exhibit significant chemical plasticity, which may explain its multiple biological effects. In this context, it is also relevant to justify the choice of solvent and extraction method used in this study based on previous investigations describing the wound healing or anti-inflammatory effects of 96% ethanolic extracts of *A. cordifolia* leaves. However, such studies did not chemically characterize the alcoholic extracts used in the experiments [10, 41, 42]. Given the similarity in the extraction methods, the extracts used in the reference studies were also considered to be rich in phytol.

As the extract produced in this study exhibited a high concentration of phytol, it was relevant to examine the potential biological and pharmacological properties of this molecule that could help explain the phytotherapeutic and phytogenomic actions of *A. cordifolia*, related to its wound healing properties. Phytol is a diterpenic, unsaturated, and long-chain acyclic alcohol that produces molecules derived from phytanic acid (PA) and is abundant in nature. This is because it is a constituent of chlorophyll, which is produced by all photosynthetic organisms, including algae, plants, and cyanobacteria. However, as humans cannot digest the chlorophyll in their diet, the primary source of phytol is ruminant-derived products, primarily meat and milk. Evidence has described numerous relevant biological properties of phytol, including antioxidant, anti-inflammatory, antimicrobial, neuroprotective, and antidiabetic effects [43, 44].

However, the genotoxic potential of phytol and of plants rich in this component remains relatively unclear, making it important to investigate the safety of using extracts from plants rich in this molecule. This statement emphasizes the significance of the results obtained in the first phase of the study, which aimed to assess the effect of *A. cordifolia* extract on viability and genotoxicity in fibroblasts.

To this end, an initial noncellular assay was conducted to identify the genotoxic and genoprotective effects of *A. cordifolia* alcoholic extract. A noncellular assay used as a reference in pharmacognostic studies is the DPPH assay (2,2-diphenyl-1-picrylhydrazyl), which evaluates the antioxidant capacity of chemical compounds, natural substances, and plant extracts. Antioxidant capacity is quantified by measuring the reduction of DPPH, which is directly proportional to the antioxidant capacity of the tested compound. However, although widely used, the antioxidant capacity of a specific extract does not necessarily imply that its action is beneficial under biological conditions (*in vivo* or *in vitro*), because the cellular redox balance is finely regulated. Increased antioxidant activity can also induce redox stress, leading to cell dysfunction and cytotoxicity [45].

However, directly investigating the cytogenotoxic action of a given extract in *in vitro* models can be time-consuming and costly until the potential therapeutic concentration range is adequately identified. In this context, our research team described and validated a noncellular method to assess the genotoxic and/or genoprotective capacity of extracts or isolated molecules by exposing pure dsDNA molecules to H_2_O_2_ and in those without its addition [13].

Therefore, the results obtained in the GEMO assay revealed that concentrations between 0.1 and 1 *µ*g/mL were candidates for presenting relevant biological activity in experimental models *in vitro*. The *in vitro* results confirmed that concentrations below 10 *µ*g/mL did not affect the viability of fibroblasts, and thus, did not induce an increase in apoptosis rates. Given that *A. cordifolia* is commonly used for open skin lesions, despite its healing action, there might be some level of genotoxic and potentially carcinogenic effects that users are unaware of. Therefore, this study evaluated the effects of the extract on the frequency of MN and other nuclear alterations in fibroblast cultures.

The results, particularly those obtained in the analysis of MN frequency, are considered relevant, as this assay is recommended by international organizations, such as the International Atomic Energy Agency, the Organization for Economic Co-operation and Development, and the International Organization for Standardization. Assessing the frequency of MN frequency, which are composed of small chromatin-containing rounded bodies visible in the cytoplasm, is relevant because these structures are caused by DNA damage or genomic instability. Currently, most factors leading to MN occurrence are well-known and have been described in the literature [46]. In the literature review, no prior study was found to have assessed the *A. cordifolia* extract for its protective effect on MN frequency at concentrations less than 1 *µ*g/mL. However, it is essential to note that a previous study conducted by Villegas et al. [47], using the Ames test with *Salmonella* typhimurium, did not find any mutagenic activity in the *A. cordifolia* extract, reinforcing the results described here.

In the MN assay, the frequency of nuclei in apoptosis was counted and compared with the control and was found to be higher in cultures exposed to *A. cordifolia* extract. However, these results are not entirely negative because the induction of apoptosis in cells with some DNA damage is essential for reducing potential carcinogenic events [48]. Based on these results, the concentration of 1 *µ*g/mL of the extract was chosen to assess the effect of *A. cordifolia* on fibroblast cultures in which an injury was mimicked using a scratch assay.

In this study, we investigated the effect of *A. cordifolia* on lesions of fibroblasts cultured in normoglycemic and hyperglycemic media, to mimic the conditions in diabetes. To better interpret the obtained results, it is essential to highlight that the wound healing process involves four main phases: (1) hemostasis/coagulation, (2) recruitment of fibroblasts and inflammatory cells, (3) proliferative phase, and (4) maturation phase. The final phase triggers the re-epithelialization process induced by deposition of the extracellular matrix (ECM), primarily through collagen production by fibroblasts. Furthermore, types III and IV collagen fibers reorganize, such that the tissue is remodeled and neovascularized [49].

In this context, the effect of *A. cordifolia* on the rate of fibroblast migration to the injury site, a phenomenon that occurs during the first phase of wound healing, was investigated. The results showed similar outcomes between fibroblasts exposed to the extract and those in the control at 6 hr after the monolayers were scratched. However, after 24 hr, the migration rate of fibroblasts exposed to *A. cordifolia* was significantly higher than that in the control. After 6 and 24 hr, the migration rate of fibroblast monolayers cultured under hyperglycemic conditions was lower. The accelerating effect on the migration rate of scratched dermal fibroblasts was similar to a study conducted on scratched 3T3 embryonic fibroblasts using a hydroalcoholic extract of *A. cordifolia* [50]. The main difference between the results obtained in this study and those described by these authors is that the higher migratory proliferative capacity described by Hanafiah et al. [50] was achieved at a much higher extract concentration (62.5 *µ*g/mL). This difference may be related to elevated phytol levels in the tested extracts. This assumption is corroborated by previous studies on other phytol-rich plants that have described their therapeutic effects in wound healing [51, 52, 53].

In the presence of *A. cordifolia*, the deceleration in fibroblast migration caused by increased glucose levels was reversed, similar to that in the control cultures. Unlike wound healing in healthy individuals, hyperglycemic conditions in patients with diabetes directly affect this process, especially in the first phase, during which there is an increase in proinflammatory cytokines, proteases, and reactive oxygen species (ROS). Additionally, this delay can occur because of cellular dysfunction triggered by hyperglycemia [54].

The pro-migratory effects of fibroblasts on the injured region may be related to the previously reported antioxidant and anti-inflammatory properties of *A. cordifolia* extracts [41, 55, 56].

It is well-known that in addition to cells, body tissues are composed of the extracellular matrix, which plays a critical role in embryogenesis, adult development, tissue and organ homeostasis maintenance, and tissue repair. Extracellular matrix components, such as collagen, are primarily synthesized by fibroblasts and subsequently secreted into the extracellular space, where they undergo modifications to become the final molecules of their organization [57]. In this context, the effect of *A. cordifolia* extract on the markers of extracellular matrix metabolism was also investigated, particularly the concentration of collagen secreted outside the cell and the expression of three genes that are modulated during the proliferative phase of wound healing (COL-1A, FGF-7, and MMP-1).

Compared with the control group, fibroblasts exposed to *A. cordifolia* exhibited increased collagen levels and overexpression of COL1-A and FGF-7. These results corroborate the traditional use of this plant in wound healing and suggest a direct phytogenomic effect on collagen metabolism and cell proliferation. This is significant as these genes are relevant in wound healing and forming functional scars [49, 58]. In contrast, *A. cordifolia* does not modulate the expression of metalloproteinases, such as MMP-1.

In a hyperglycemic environment, *A. cordifolia* extract also induced a higher rate of collagen secretion than in the control cultures, along with the overexpression of COL-1A and FGF-7 genes. Delayed or impaired wound healing affects ~25% of patients with diabetes. Studies on diabetic foot wounds have shown altered fibroblast morphology, lack of growth factors, reduced migration rates, proliferation, and decreased extracellular matrix deposition [59]. Previous studies have also described the potential antidiabetic effects of *A. cordifolia*, as seen in Dwitiyanti et al. [12] and Sulfianti et al. [60]. Furthermore, a previous study reported that 10% and 30% alcohol-based *A. cordifolia* gel effectively healed diabetic wounds [11, 61].

## 5. Conclusion

Despite the methodological limitations inherent to *in vitro* studies, our results are relevant as they point to a pro-healing effect of the ethanolic extract of *A. cordifolia* in normoglycemic and hyperglycemic environments. The plant presented an antioxidant, genoprotective, and nongenotoxic effect at low concentrations, and the extract was able to increase collagen concentration in the supernatant in a model of scratched fibroblasts cultivated in a hyperglycemic environment. *A. cordifolia* extract differentially modulated gene expression related to the formation of the extracellular matrix in scratched fibroblasts, increasing the expression of genes related to the healing process. More studies need to be carried out to better understand the causal mechanisms of the plant's effects, so that we can seek the synthesis of new medicines that can contribute to the healing of wounds, especially those that are difficult to manage, such as diabetic wounds.

## Figures and Tables

**Table 1:** Primer sequences.

Gene	Functional relevance in the wound healing processes	Primers
MMP-1	Matrix metalloproteinase 1 gene Collagenase enzyme activated in the inflammatory phase and in the remodeling phase, replacing damaged tissue and forming a mature and functional scar	Sense: 3'AGTGACTGGGAAACCAGATGCTGA5' Antisense: 5'TCAGTGAGGACAAACTGAGCCACA3'

COL-1A	Collagen type 1 alpha 1 chain gene Essential for effective wound healing and the formation of functional scars	Sense: 3'TCTTGGTCAGTCCTATGCGGATA5' Antisense: 3'CATCGCAGAGAACGGATCCT

FGF-7	Fibroblast growth factor 7: Mitogen factor specific for epithelial cells and keratinocytes involved in the proliferation promotion of dermal fibroblasts and enhance the production of extracellular matrix components	Sense: 3'CTGTCGAACACAGTGGTACCTG5' Antisense: 5'CCAACTGCCACTGTCCTGATTTC3'

*β*-Actin	A cytoskeletal protein found in all eukaryotic cells, used to normalize the expression of genes related to wound healing	Sense: 3'TGTGGATCAGCAAGCAGGAGT5' Antisense: 5'TGCGCAAGTTAGGTTTTGTCA3'

**Table 2:** Chemical matrix components of *A. cordifolia* leaves described in the literature according to solvent.

Solvent	Main bioactive molecules	References
Crude ethanolic extract partially with *n*-hexane	Triterpenoid sapogenins: larreagenin A, 3-hydroxy-30-noroleana-12,20(29)-dien-28-oic acid, ursolic acid, 28-ethyl 30-methyl (3*β*)-3,23-dihydroxyolean-12-ene-28,30-dioate	[27]
Methanol	Boussingoside (A1, A2, B, and C), momordin, and larreagenin A [16]	[28]
Hydroalcoholic extract (70%)	Bougracol A, 4,7-dihydroxy-5-methoxy-8-methyl-6-formyl-flavane, 7-O-methylunonal, 5,7-dihydroxy-6,8-dimethyl-2-phenyl-4H1-benzopyran-4-one, desmosflavone, and demethoxymatteucinol	[29]
Methanol extract	8-Glucopyranosyl-4',5,7-trihydroxyflavone	[30]
Ethanol	Alkaloid bethanidine, p-coumaric acid	[31, 32]
Crude ethanolic extract	—	[33]
Ethanolic extract	Phytol and *n*-hexadecanoic acid	[34]
Hydroalcoholic extract	Saponins, flavonoids, and alkaloids (steroids/triterpenoids)	[35]
Crude ethanolic extract partially with ethyl acetate	Flavonoids orientoside	[36]
Aqueous methanol (70%)	3-Hydroxy-alpha-ionone	[37]
Different extraction methods	Alkaloids, flavonoids, saponins, tannins, steroids, and phenolic compounds, specially Azedarachin C and Vitexin	[38]
Ethanolic extract (60%)	Flavonoids, steroids, phenols, alcoholics, terpenoids, and saponins. The GC–MS analysis found high concentration of phytol and containing fatty acids	[9]
Ethanolic extract (96%)	Phenolic compounds, flavonoids, alkaloids, and tannins	[39]
Methanol	Total phenolic content	[6]

## Data Availability

The data used to support the findings of this study are available from the corresponding author upon request.
